# Comparison of the Effectiveness of Interscalene Nerve Block and Serratus Posterior Superior Intercostal Plane Block in Patients Undergoing Arthroscopic Shoulder Surgery

**DOI:** 10.3390/healthcare14081028

**Published:** 2026-04-14

**Authors:** Omer Doymus, Ela Nur Medetoglu, Habip Burak Ozgodek, Ozlem Dilara Erguney, Pelin Aydın, Nasuhi Altay, Aslı Turgut, Ali Ahiskalioglu

**Affiliations:** 1 Department of Anaesthesiology and Reanimation, Erzurum City Hospital, 25070 Erzurum, Turkey; brk.ozgodek@gmail.com (H.B.O.); ozlemdilaraaydin@hotmail.com (O.D.E.); dr.paydin@hotmail.com (P.A.); 2 Department of Anaesthesiology and Reanimation, Oltu State Hospital, 25400 Erzurum, Turkey; elamdtoglu@gmail.com; 3 Department of Orthopedics and Traumatology, Erzurum City Hospital, 25070 Erzurum, Turkey; onasuhialtay@hotmail.com; 4 Department of Radiology, Erzurum City Hospital, 25070 Erzurum, Turkey; asli.turgut.asli@gmail.com; 5 Department of Anesthesiology and Reanimation, Faculty of Medicine, Ataturk University, 25240 Erzurum, Turkey; aliahiskalioglu@hotmail.com

**Keywords:** interscalene brachial plexus block, serratus posterior superior intercostal plane block, shoulder arthroscopies

## Abstract

**Highlights:**

**What are the main findings?**

- Shoulder arthroscopies are painful procedures, and interscalene block, an analgesic technique, can impair respiratory function.
- Various regional anesthesia techniques continue to be developed to avoid opioid side effects and pulmonary complications.

**What are the implications of the main findings?**

- Whereas the interscalene brachial plexus block efficiently manages pain during shoulder arthroscopic procedures, the serratus posterior superior intercostal plane block is seen as a more beneficial treatment regarding respiratory safety.
- More randomized controlled trials are needed to better evaluate the effectiveness of the block.

**Abstract:**

**Background/Objectives**: Shoulder arthroscopies are commonly conducted in orthopedic practice. The interscalene brachial plexus block (ISB) is regarded as the “gold standard” for postoperative analgesia in shoulder surgeries. The serratus posterior superior intercostal plane block (SPSIPB) was introduced as an innovative treatment for addressing thoracic and shoulder discomfort. This study aims to examine the effects of SPSIPB and ISB techniques on postoperative pain levels, opioid intake, and respiratory function measures in patients having shoulder arthroscopy. **Methods**: Patients were divided into two groups. In the ISB group, 15 mL of fluid containing 0.25% bupivacaine was applied between interscalene muscles, while in the SPSIPB group, 30 mL of 0.25% bupivacaine was applied in the fascial plane between the serratus posterior superior muscle and the intercostal muscles. **Results**: There were no statistically significant differences in demographic characteristics (*p* > 0.05). VAS scores were statistically lower in the ISB group compared to the SPSIPB group at rest at 1, 2, 4, 8, 12, and 24 h postoperatively in the PACU (*p* < 0.05). VAS scores were also lower in the ISB group compared to the SPSIPB group during active movement at 1, 2, 4, 8, and 12 h postoperatively in the PACU (*p* < 0.05). Twenty-four-hour fentanyl consumption was lower in the ISB group compared to the SPSIPB group (407.50 ± 169.32 μg and 767.50 ± 178.00 μg, respectively, *p* < 0.001). The decrease in FEV_1_ and FVC was higher in the ISB group compared to the SPSIPB group (*p* < 0.001). **Conclusions**: ISB effectively relieves pain during shoulder arthroscopic procedures; however, while SPSIPB is considered a more advantageous option in terms of respiratory safety, it may not provide adequate analgesia on its own.

## 1. Introduction

Shoulder arthroscopies, commonly conducted in orthopedic practice, are operations linked to considerable postoperative pain despite their minimally invasive nature. A considerable percentage of individuals undergoing a shoulder arthroscopy (up to 45%) experience intense pain during the early postoperative phase [1]. In this cohort, proficient care of postoperative pain is essential for enhancing patient satisfaction, expediting the rehabilitation process, and reducing discharge time [2]. Consequently, regional anesthesia is extensively employed in multimodal analgesia protocols to mitigate opioid usage and its related adverse effects [3,4].

The interscalene brachial plexus block (ISB) is regarded as the “gold standard” for postoperative analgesia in shoulder surgeries and is the most commonly utilized technique in clinical practice [2,3]. While ISB offers good analgesia in the shoulder area and diminishes perioperative opioid usage in patients, its primary drawback is the induction of phrenic nerve block resulting from its anatomical proximity. The ipsilateral phrenic nerve block that may occur with an interscalene block might result in hemidiaphragmatic paralysis (HDP) [4,5,6].

The phrenic nerve block, which may occur alongside the interscalene block, results in compromised diaphragm functionality. This leads to decreases in respiratory metrics such as forced vital capacity (FVC) and forced expiratory volume in 1 s (FEV_1_) by 25–30%, with certain studies indicating reductions of up to 40%. Although these declines in respiratory function are typically manageable in healthy individuals, they pose significant risks, including severe dyspnea, respiratory failure, and hypoxia, in patients with limited respiratory reserve, such as those with chronic obstructive pulmonary disease, morbid obesity, or advanced age [7,8,9].

These dangers have prompted practitioners to devise alternative “diaphragm-sparing” localized approaches. While techniques like suprascapular nerve block and superior trunk block are employed for this purpose, research indicates that in comparison to interscalene block (ISB) these methods may exhibit diminished efficacy in achieving comprehensive analgesia of the shoulder area and may not entirely mitigate the risk of developing hemidiaphragmatic paralysis (HDP) [1,8,9,10]. Consequently, there is a necessity to devise novel and alternative methodologies in shoulder procedures that can deliver effective analgesia while concurrently safeguarding respiratory function.

The serratus posterior superior intercostal plane block was introduced by Tulgar et al. as an innovative treatment for addressing thoracic and shoulder discomfort [11]. Clinical applications indicate that SPSIPB offers an extensive sensory blockade from C3 to T10 levels, resulting in effective analgesia in the shoulder, axilla, hemithorax, and posterior neck [6,11]. The anatomical positioning of the SPSIPB application location, being more distal and posterior to the brachial plexus and phrenic nerve, indicates that this block may serve as a safer analgesic method in shoulder surgery, preserving respiratory function [11,12].

The primary aim of this study was to examine the effects of SPSIPB and ISB techniques on opioid consumption, and the secondary aim was to examine their effects on postoperative pain levels and respiratory function measures and to investigate if SPSIPB is determined to be an efficient diaphragm-sparing analgesic option.

## 2. Materials and Methods

This prospective randomized controlled trial, approved by the ethics committee (Yüzüncü Yıl University, Van, Turkey, 04.11.2024-B.30.2.YYU.0.01.00.00/92, ClinicalTrials.gov NCT07311096), included 60 participants aged 18–65 years in the ASA I-III classification scheduled for unilateral elective arthroscopic shoulder surgery and with no history of allergy to the medications to be used, no chronic pain, and no history of analgesic use in the last 24 h. Patients eligible for the trial were apprised of its details. Patients who declined participation, those allergic to the medications to be administered, and individuals with a history of severe systemic disease were excluded from the study. Patients were randomly allocated to two equal groups with the Microsoft Excel RAND function and Envelope Method to receive either the ISS block or the SPSIP block. To mitigate performance bias, all blocks were conducted by a singular experienced anesthesiologist. All other researchers, assessors and patients were unaware and blinded to the group allocation.

### 2.1. Interscalene Block Procedure

Patients were summoned to the operating room one hour prior to operation without any premedication. Both patient cohorts received 1–2 mg of intravenous midazolam for sedation in the block room. Prior to the interscalene block, an anatomical scan was conducted with a linear (L15-4) ultrasound probe (Piloter™ Ultrasound System, Wisonic Medical Technology Co., Ltd., Shenzhen, China) to ascertain the position of the brachial plexus. Upon positioning the USG probe in the supraclavicular region, the C5 and C6 nerve roots were seen between the anterior and middle scalene muscles through an upward scanning technique. Following skin sterilization, infiltration was conducted using 1 cc of 1% lidocaine. An interscalene block was administered, utilizing a 22G 50 mm needle with ultrasound and neurostimulator guidance through a posterior in-plane technique. The needle tip location was identified via ultrasound. After the needle tip was situated just lateral to the brachial plexus sheath and next to the C5 and C6 nerve roots, 15 mL of 0.25% bupivacaine was administered.

### 2.2. SPSIP Block Application

Patients were seated, and flexion, adduction, and internal rotation were administered to the upper arm on the targeted side to lateralize the scapula, as delineated by Tulgar et al. [11]. Upon positioning a high-frequency linear (L15-4) ultrasound probe (Piloter™ Ultrasound System, Wisonic Medical Technology Co., Ltd., Shenzhen, China) at the level of the scapular spines in the transverse plane, the upper medial border of the scapula, trapezius muscle, rhomboid muscle, serratus posterior muscle, and the second and third ribs were discerned. The ultrasound probe was turned 90 degrees from the posterior aspect of the supraclavicular fossa to enhance visualization of the first rib. Following the identification of the first rib, the second and third ribs were further verified. Following skin sterilization, infiltration was conducted with 1 cc of 1% lidocaine. A 22G 8 mm block needle was placed medially to the scapula using an in-plane method, targeting the fascial plane between the serratus posterior superior muscle and the intercostal muscles. Following the verification of the position with 2 mL of isotonic solution, 30 mL of 0.25% bupivacaine was administered. Following the application of the blocks, the dermatomes were assessed utilizing a hot/cold skin test.

### 2.3. Diaphragmatic Motions

**Figure 1:**
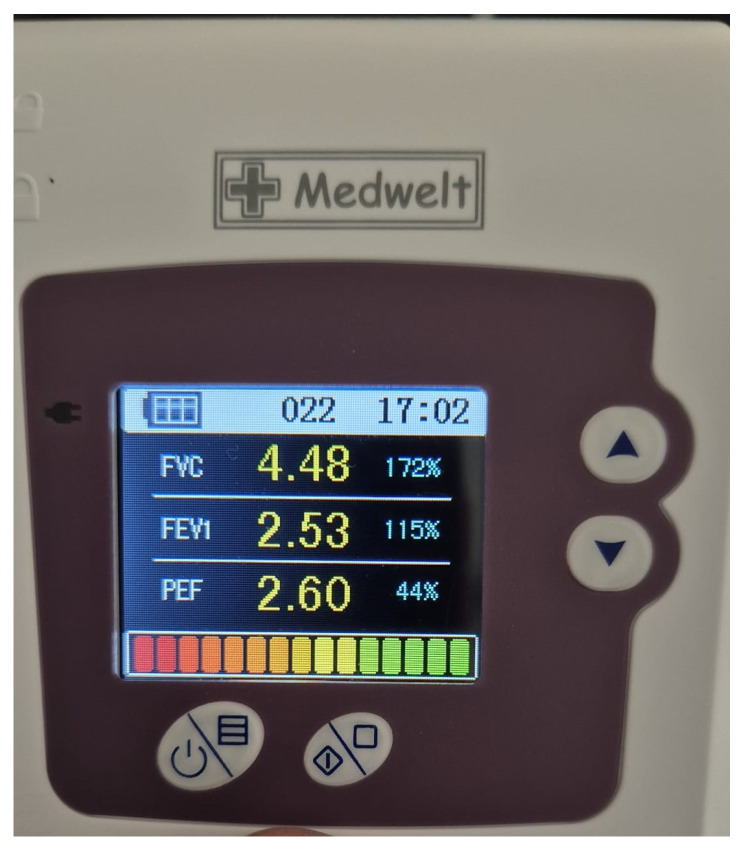
Spirometric values obtained with a Medwelt Spiromter SP10.

Ipsilateral diaphragmatic excursion was evaluated in both groups before block and 30 min post-block by an anesthesiologist blinded to group allocation, utilizing M-mode ultrasonography in a seated posture. Following the identification of the lowest rib in the anterior-middle axillary line using a convex (C5-2) probe (Piloter™ Ultrasound System, Wisonic Medical Technology Co., Ltd., Shenzhen, China), the spleen or liver served as an acoustic window and diaphragmatic excursion between inspiration and expiration was quantified in centimeters. Each test was conducted thrice, and the mean values were documented. The intensity of hemidiaphragmatic paresis was quantified by the reduction in diaphragmatic excursion, expressed as a percentage difference, between baseline and 30 min post-block completion. A reduction in diaphragmatic movement exceeding 75% from baseline, immobility, or paradoxical movement was classified as complete paresis; a reduction between 25% and 75% was classified as partial paresis; and a reduction of less than 25% was classified as no paresis [13]. Hemidiaphragmatic paresis was deemed present if either partial or total paresis was observed. Lung function tests were assessed prior to and 30 min subsequent to the block, utilizing a bedside spirometer (Medwelt Spiromter SP10, Contec Medical Systems Co., Ltd., Qinhuangdao, Hebei Province, China) with the patient seated. Spirometry was conducted thrice with the patient seated, and the mean of the measurements was recorded. Forced expiratory volume in one second (FEV_1_) and forced vital capacity (FVC) were measured thrice, and the average values were recorded. The test was deemed accurate when the screen indicator went green (Figure 1).

### 2.4. General Anesthesia

Upon concluding post-block evaluations, all patients underwent orotracheal intubation utilizing intravenous propofol (1.5–2.0 mg·kg^−1^), rocuronium (0.6–0.8 mg·kg^−1^), and intravenous fentanyl (2 μg·kg^−1^·min^−1^). Anesthesia was sustained using sevoflurane in a mixture of air and oxygen. Patients were administered a remifentanil infusion at a rate of 0.01 to 0.1 μg·kg^−1^·min^−1^. Patients were relocated to the post-anesthesia care unit following extubation.

### 2.5. Postoperative Analgesia Protocol

Thirty minutes before the conclusion of surgery, each patient was administered 1000 mg of paracetamol intravenously, with further doses repeated every eight hours during the postoperative period. The identical procedure for postoperative analgesia was implemented for both groups. Patients were administered intravenous patient-controlled analgesia (PCA) in the post-anesthesia care unit (PACU) for postoperative pain management. The PCA, prepared with fentanyl, was programmed at a concentration of 10 mcg/mL, with a 15 min lock-in time, a 25 mcg bolus, and no basal infusion, and continued for 24 h. In the recovery room, patients exhibiting a VAS score of 4 or above were administered an extra 25 mg of meperidine, which was duly documented. Patients exhibiting an Aldrete score of 9 or above were sent to the ward. The postoperative follow-up and assessment of the cases were both conducted by a researcher blind to the study groups. Postoperative pain evaluation was conducted in the PACU at 1, 2, 4, 8, 12, and 24 h.

### 2.6. Statistical Analysis

To decide on the required sample size, a pilot study has been done. A pilot study was conducted to determine the necessary sample size. In the pilot study, our primary target, 24-h opioid consumption (fentanyl-mcq), was found to be 545.00 ± 143.33 mcq in the ISB group (*n* = 10) and 715.00 ± 179.96 mcg in the SPSIPB group (*n* = 10). A sample size of 21 patients in total was computed for each group via G*Power version 3.1.9.2 (Heinrich Heine University Düsseldorf), with an effect size of 1.057, a power of 0.95, and an alpha probability of 0.05. Considering dropouts, it was decided that at least 60 participants would be recruited. Data was analyzed using SPSS Statistics 22 software (IBM, Armonk, NY, USA). Following assessment for normal distribution with the Kolmogorov–Smirnov test, the normal distributing data were analyzed with Student’s *t*-test, and non-normally distributed data were evaluated using the Mann–Whitney U test. Categorical data such as the need for rescue analgesic, complications, adverse events were assessed using Chi-square tests and Mann–Whitney U or Student *t*-tests for continuous measures. Statistical significance was accepted when *p* < 0.05. All *p* values were calculated as two-sided.

## 3. Results

**Figure 2:**
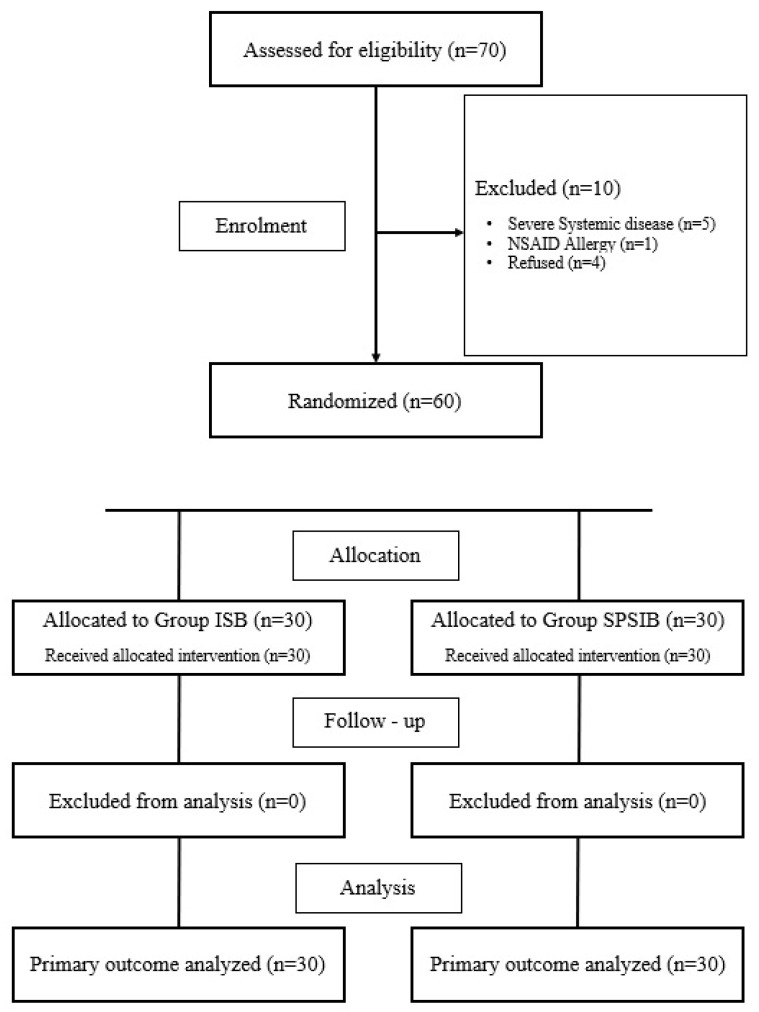
Flow diagram of the study.

After excluding 10 out of the 70 eligible patients, a total of 60 patients were randomly assigned to two different groups (Figure 2). The patient age was 48.5 ± 13.5 years in the ISB group and 51.7 ± 13.1 years in the SPSIPB group. The patient weight was 77.8 ± 9.0 kg in the ISB group and 78.7 ± 10.7 kg in the SPSIPB group. There were no statistically significant differences between the groups in terms of weight, age, height, duration of the anesthesia or the duration of the surgery (*p* > 0.05). Detailed results were reported in Table 1.

The VAS scores were also significantly higher during rest in the at PACU, 1, 2, 4, 8, 12 and 24 h postoperatively in the SPSIPB group than in the ISB group (*p* < 0.05). The VAS scores were also significantly higher during active movement in the at PACU, 1, 2, 4, 8 and 12 h postoperatively in the SPSIPB group than in the ISB group (*p* < 0.05). The VAS scores during active movement and resting were similar in both groups at 24 h (*p* > 0.05) (Table 2 and Table 3).

The 24 h postoperative fentanyl consumption was 407.50 ± 169.32 µg in the ISB group and 767.50 ± 178.00 µg in the SPSIPB group, and the difference was found to be statistically significant (*p* < 0.001) (Table 4). The number of patients requiring rescue analgesia was 7 in the ISB group and 28 in the SPSIPB group (*p* < 0.001). No statistically significant differences between the groups were observed in terms of side effects (*p* > 0.05) (Table 5).

Statistically significant differences were found between the groups in terms of diaphragm movement difference (*p* < 0.001), FEV_1_ difference (*p* < 0.001), and FVC difference (*p* < 0.001). In all parameters, the ISB group’s values were significantly higher than the SPSIPB group’s (Table 6).

## 4. Discussion

This study compared interscalene brachial plexus blocks, which target the brachial plexus roots at the interscalene level, with serratus posterior superior intercostal plane blocks, which are applied to the fascial plane between the intercostal muscles and the serratus posterior superior muscle, regarding postoperative pain, opioid consumption, diaphragmatic function, and pulmonary parameters in patients undergoing arthroscopic shoulder surgery. Our findings indicate that patients undergoing arthroscopic shoulder surgery with interscalene block (ISB) had reduced postoperative pain levels and diminished opioid usage in comparison to single serratus posterior superior intercostal plane block (SPSIPB). Nonetheless, whereas ISB offered enhanced analgesia relative to SPSIPB, it resulted in considerable deterioration in diaphragmatic function and pulmonary metrics. These findings underscore the essential equilibrium between analgesic effectiveness and respiratory safety in ISB and SPSIPB regional anesthetic methods employed in shoulder surgery. In our investigation, postoperative VAS ratings were assessed at rest and during movement. VAS scores were found to be considerably lower in the ISB group than in the SPSIPB group at nearly all time intervals within the initial 24 h. This finding can be elucidated by the ability of ISB to establish a potent blockade on the C5–C6 nerve roots, which are integral to the sensory innervation of the shoulder joint [5].

A literature analysis demonstrates that randomized clinical trials and analyses confirm that ISB remains the most efficacious regional method for postoperative pain management following shoulder arthroscopy [2]. The superior analgesic performance of ISB can be explained by its direct blockade of the C5–C6 nerve roots, which contribute substantially to the suprascapular and axillary nerves—key components of the sensory innervation of the glenohumeral joint. This targeted neural blockade allows ISB to effectively suppress deep intra-articular nociceptive input, which is the predominant source of pain following arthroscopic shoulder procedures [14]. In contrast, the inferior analgesic efficacy observed with SPSIPB appears to be primarily related to its anatomical and mechanistic limitations. Unlike ISB, SPSIPB does not directly target the major articular nerves of the shoulder. Instead, it relies on the spread of local anesthetic within a fascial plane to achieve indirect and predominantly cutaneous or posterior thoracic sensory blockade [6,11]. In this investigation, SPSIPB proved inadequate in alleviating intra-articular shoulder pain. Importantly, although interindividual variability in local anesthetic spread within fascial planes may contribute to differences in block performance, this factor alone is unlikely to explain the magnitude and consistency of the observed differences in both pain score and opioid consumption [15]. Therefore, in the present study, the limited analgesic efficacy of SPSIPB should be interpreted primarily as a consequence of insufficient coverage of the suprascapular and axillary nerve territories, rather than variability in anesthetic spread. In our investigation, total fentanyl intake over 24 h and the requirement for supplementary analgesia were considerably elevated in the SPSIPB group, aligning with pain scores. The diminished opioid necessity noted in the ISB group reinforces the notion that interscalene brachial plexus block is an efficacious regional anesthesia technique that conserves opioids in multimodal analgesia approaches for shoulder surgery [1,2]. From a conceptual standpoint, it is also important to recognize that ISB and SPSIPB are not anatomically or functionally equivalent techniques. ISB is a well-established, targeted nerve block, whereas SPSIPB represents a relatively novel interfascial plane block with a different mechanism of action. Accordingly, the findings of this study should not be interpreted as a comparison between two equivalent analgesic strategies, but rather as an evaluation of SPSIPB in relation to ISB as a reference standard.

We evaluated both groups for side effects and found no statistically significant difference in opioid-related adverse effects, such as nausea, vomiting, pruritus, and urine retention. Our investigation revealed that opioid usage was elevated in the SPSIPB group. We think that using the same pain management methods and patient-controlled analgesia relief in both groups may have kept the side effects at similar levels. This suggests that the variance in opioid dosage alone does not dictate the emergence of side effects; rather, the analgesic method employed and adjunctive therapies used significantly influence the side effect profile. Our data clearly show that we can achieve a manageable side effect profile in shoulder surgery by using the right multimodal analgesia strategies, even if the amount of opioids used varies. Comparable results in the literature corroborate our findings [2,16].

Despite its inferior analgesic efficacy, SPSIPB demonstrated a clear advantage in terms of respiratory safety. The significantly smaller reductions in diaphragmatic excursion, FEV_1_, and FVC observed in the SPSIPB group are consistent with its anatomical distance from the phrenic nerve. In contrast, ISB is well known to cause ipsilateral hemidiaphragmatic paresis due to its proximity to the phrenic nerve, even when performed with reduced volumes [17]. These findings highlight the well-recognized trade-off between optimal analgesia and preservation of respiratory function in regional anesthesia for shoulder surgery. This conclusion aligns with other research indicating that ISB often results in ipsilateral hemidiaphragmatic paralysis due to its physical closeness to the phrenic nerve. A 25–40% reduction in diaphragmatic movement has been documented following ISB, accompanied by clinically substantial declines in FEV_1_ and FVC values. Riazi et al. asserted that, despite utilizing low-volume ISB procedures under ultrasound guidance, total prevention of phrenic nerve blockage was unattainable, resulting in ongoing respiratory function deterioration [7]. A comparable study also indicated a significant occurrence of hemidiaphragmatic paralysis following ISB and its impact on pulmonary function assessments [9]. The reduction in diaphragmatic mobility and spirometric metrics noted in the ISB group in our study substantially aligns with existing literature findings. This reiterates the detrimental effect of ISB on respiratory functioning, notwithstanding its high analgesic efficacy. Upon analyzing the SPSIPB group, we noted no substantial alterations in diaphragmatic excursion, FEV_1_, and FVC measurements. We determined that respiratory function was predominantly maintained. A substantial segment of the case series concerning SPSIPB was conducted after thoracic and scapular procedures, and these investigations did not document phrenic nerve involvement or clinically severe diaphragmatic paralysis [18]. Comparable results from our study of patients having shoulder arthroscopy indicate that the diaphragm-sparing characteristic of SPSIPB may also be relevant in shoulder surgery. The posterior and distal injection site of SPSIPB, located anatomically away from the phrenic nerve, along with the diffusion of the local anesthetic throughout the fascial plane account for the limited impact of this block on diaphragm function [11,12]. The clinical implications of these findings should therefore be interpreted with caution. While SPSIPB may offer a diaphragm-sparing advantage, its significantly inferior analgesic profile precludes it from being considered a general alternative to ISB for arthroscopic shoulder surgery.

Our study had some limitations. Pulmonary function tests were performed by the study team rather than specialized respiratory technicians, which may have introduced measurement variability. Additionally, the study population consisted predominantly of middle-aged patients with relatively preserved baseline pulmonary function, which may limit the generalizability of the respiratory findings to higher-risk populations.

## 5. Conclusions

In summary, whereas the interscalene brachial plexus block delivers enhanced analgesia for arthroscopic shoulder procedures, the serratus posterior superior intercostal plane block may confer benefits in maintaining respiratory function. However, due to its somewhat limited analgesic efficacy, the effectiveness of SPSIPB may be limited to carefully selected patients in whom respiratory failure is a significant problem and whose reduced analgesic efficacy is clinically tolerable or can be supported by additional analgesic strategies, rather than as a standard alternative. Consequently, the selection of the block approach must be personalized, considering the patient’s clinical attributes and the perioperative respiratory risk. Future research should focus on optimizing the role of SPSIPB within multimodal analgesia protocols or in combination with other regional techniques to improve its analgesic efficacy, rather than evaluating it as a standalone alternative to ISB.

## Figures and Tables

**Table 1:** Comparison of demographic data.

	Group ISB (*n*:30)	Group SPSIPB (*n*:30)	*p*
Gender (F/M)	13/17	14/16	0.795 ^b^
Age (years) (mean ± SD)	48.5 ± 13.5	51.7 ± 13.1	0.260 ^a^
Weight (kg) (mean ± SD)	77.8 ± 9.0	78.7 ± 10.7	0.807 ^a^
Height (cm) (mean ± SD)	167.3 ± 9.7	166.0 ± 8.4	0.534 ^a^
Duration of the surgery (mean ± SD)	72.8 ± 19.6	65.8 ± 15.8	0.256 ^a^
Duration of the anesthesia (mean ± SD)	96.6 ± 19.4	89.2 ± 16.9	0.157 ^a^

Values are expressed as mean ± standard deviation; BMI, body mass index. ^a^ Mann–Whitney U test between groups. ^b^ Chi-square test between groups.

**Table 2:** VAS scores in resting.

	Group ISB (*n*:30)	Group SPSIPB (*n*:30)	*p*
VAS PACU	0 (0–0)	4 (2–6)	<0.001 ^a^
VAS 1st h	0 (0–1)	4 (3–5)	<0.001 ^a^
VAS 2nd h	0 (0–2)	4 (3–5)	<0.001 ^a^
VAS 4th h	1 (0–2)	3.5 (3–4)	<0.001 ^a^
VAS 8th h	1 (0–2)	3 (2–4)	<0.001 ^a^
VAS 12th h	2 (1–2)	3 (2–4)	<0.001 ^a^
VAS 24th h	2 (1–3)	3 (2–4)	0.010 ^a^

Values are given as median (IQR). PACU: Postanesthetic Care Unit. ^a^ Mann–Whitney U test between groups.

**Table 3:** VAS scores during action.

	Group ISB (*n*:30)	Group SPSIPB (*n*:30)	*p*
VAS PACU	0 (0–0)	4 (3–5)	<0.001 ^a^
VAS 1st h	0 (0–1)	4 (4–4)	<0.001 ^a^
VAS 2nd h	1 (0–2)	4 (3–4)	<0.001 ^a^
VAS 4th h	2 (1–3)	4 (3–4)	<0.001 ^a^
VAS 8th h	2 (1–3)	4 (3–5)	<0.001 ^a^
VAS 12th h	2 (2–3)	3 (3–4)	<0.001 ^a^
VAS 24th h	3 (2–3)	4 (3–4)	0.057 ^a^

Values are given as median (IQR). PACU: Postanesthetic Care Unit. ^a^ Mann-Whitney U test between groups.

**Table 4:** Fentanyl consumption (µg) via patient-controlled analgesia.

	Group ISB (*n*:30)	Group SPSIPB (*n*:30)	*p*
0 to 4 h (µg)	42.5 ± 58.03	164.16 ± 48.54	<0.001 ^a^
4 to 8 h (µg)	90.83 ± 77.26	248.33 ± 97.58	<0.001 ^a^
8 to 24 h (µg)	265.00 ± 142.87	351.66 ± 135.33	0.019 ^b^
24 h total (µg)	407.50 ± 169.32	767.50 ± 178.00	<0.001 ^b^

Values are expressed as mean ± standard deviation, ^a^ Mann–Whitney U test between groups. ^b^ Sample *t* test.

**Table 5:** Need for rescue analgesics and side effects.

	Group ISB (*n*:30)	Group SPSIPB (*n*:30)	*p*
Need for Meperidine	7 (23.3%)	28 (93.3%)	<0.001 ^b^
Nausea	17 (56.7%)	15 (50.0%)	0.605
Vomiting	7 (23.3%)	6 (20.0%)	0.098
Need for Antiemetics	1 (3.3%)	1 (3.3%)	1.000
Itching	2 (6.7%)	0 (0%)	0.492
Dry Mouth	1 (3.3%)	1 (3.3%)	1.000
Constipation	0 (0%)	0 (0%)	NS
Urinary Retention	0 (0%)	0 (0%)	NS
Block-related Complication	0 (0%)	0 (0%)	NS

Values are expressed as *n* (%), NS: Non-Significant. ^b^ Pearson Chi-square test between groups.

**Table 6:** Outcomes of diaphragmatic movement and pulmonary function between the ISB and SPSIPB group.

	Group ISB (*n*:30)	Group SPSIPB (*n*:30)	*p*
Decrease in diaphragmatic excursion	41.38	19.62	<0.001
Decrease in FEV_1_ %	40.50	20.50	<0.001
Decrease in FVC %	39.57	21.43	<0.001
FEV_1_ at baseline	2.37 ± 0.38	2.44 ± 0.63	0.994 ^a^
FVC at baseline	3.25 ± 0.86	3.09 ± 0.78	0.496 ^a^
FEV_1_ at 30 min after block	1.89 ± 0.62	2.3 ± 0.60	0.013 ^b^
FVC at 30 min after block	2.74 ± 1.00	2.96 ± 0.79	0.348 ^b^
Paralysis			
Complete/partial/none	5/15/10	0/0/30	<0.001

The values are means ± SDs or number (percentage) as appropriate. ^a^ Mann–Whitney U test between groups. ^b^ sample *t* test.

## Data Availability

The data presented in this study are not publicly available due to privacy and ethical restrictions to protect participant confidentiality.

## Abbreviations

The following abbreviations are used in this manuscript:

ISB	The interscalene brachial plexus block
HDP	Hemidiaphragmatic paralysis
FVC	Forced Vital Capacity
FEV_1_	Forced Expiratory Volume in 1 s
SPSIPB	Serratus Posterior Superior Intercostal Plane Block
